# Surgical Management of a Rare Ascending Aortic Mural Thrombus Using a Modified Brain Isolation Strategy: A Case Report

**DOI:** 10.70352/scrj.cr.25-0768

**Published:** 2026-03-27

**Authors:** Rei Sueoka, Shogo Takahashi, JeongA Lee, Akito Inoue, Kentaro Shirakura, Yuki Setogawa, Daisuke Takeyoshi, Hiroyuki Kamiya, Shingo Kunioka

**Affiliations:** Department of Cardiac Surgery, Asahikawa Medical University, Asahikawa, Hokkaido, Japan

**Keywords:** aortic mural thrombus, ECG-gated contrast-enhanced CT, unilateral selective cerebral perfusion

## Abstract

**INTRODUCTION:**

Ascending aortic mural thrombus (AMT) is an exceptionally rare condition. Diagnosis is often difficult because non-ECG-gated contrast-enhanced CT is susceptible to motion artifacts, and the distal ascending aorta can sometimes be challenging to evaluate using transesophageal echocardiography. A standardized surgical strategy for ascending AMT has not been established.

**CASE PRESENTATION:**

A 61-year-old man presented with transient left hemiplegia caused by embolic stroke. A non-ECG-gated contrast-enhanced CT scan revealed a thrombus-like structure in the ascending aorta; however, ECG-gated contrast-enhanced CT clearly demonstrated a thin-stalked and mobile thrombus, prompting urgent surgery. Cardiopulmonary bypass (CPB) was established using bilateral axillary artery cannulation and bicaval venous drainage. To minimize the risk of cerebral embolization during CPB initiation, the left common carotid artery (LCCA) was temporarily clamped under near-infrared spectroscopy monitoring. After cooling to 26°C, circulatory arrest with brachiocephalic artery and LCCA clamping and unilateral selective cerebral perfusion (uSCP) was performed. A highly mobile thrombus-like mass located just proximal to the brachiocephalic artery was excised with an adequate margin, followed by partial arch replacement with reconstruction of two arch branches. The postoperative course was uneventful, and pathology confirmed atherosclerosis with fresh thrombi and early organization.

**CONCLUSIONS:**

ECG-gated contrast enhanced CT is essential for accurate diagnosis of ascending AMT, particularly when small lesions mimic motion artifacts. The combination of bilateral axillary cannulation, LCCA occlusion, and a modified brain isolation strategy using uSCP may provide a safe and effective approach for surgical treatment of ascending AMT without neurological complications.

## Abbreviations

AMT
: aortic mural thrombus
CPB
: cardiopulmonary bypass
ECG
: electrocardiogram
LCCA
: left common carotid artery
NIRS
: near-infrared spectroscopy
SCP
: selective cerebral perfusion
TEE
: transesophageal echocardiography
uSCP
: unilateral selective cerebral perfusion

## INTRODUCTION

AMT is an uncommon clinical entity, with more than half of reported cases occurring in the abdominal aorta; only a few percentages arise in the ascending aorta, making ascending AMT exceptionally rare.^1)^ Published surgical cases are extremely scarce, and no standardized diagnostic or surgical strategy has been established for this condition.^2,3)^ Diagnostic evaluation is particularly challenging. Non-ECG-gated contrast-enhanced CT of the ascending aorta is susceptible to motion artifacts, which may obscure or mimic intraluminal lesions.^4)^ Although TEE is often considered in the evaluation of ascending aortic pathology, visualization of certain segments may occasionally be limited, making evaluation difficult in some cases.^5)^

Here, we report a rare case of ascending AMT identified after a transient ischemic neurological event. ECG-gated CT enabled precise characterization of a stalked thrombus, and semi-emergent surgery was safely performed using a brain isolation strategy with uSCP.

## CASE PRESENTATION

**Fig. 1:**
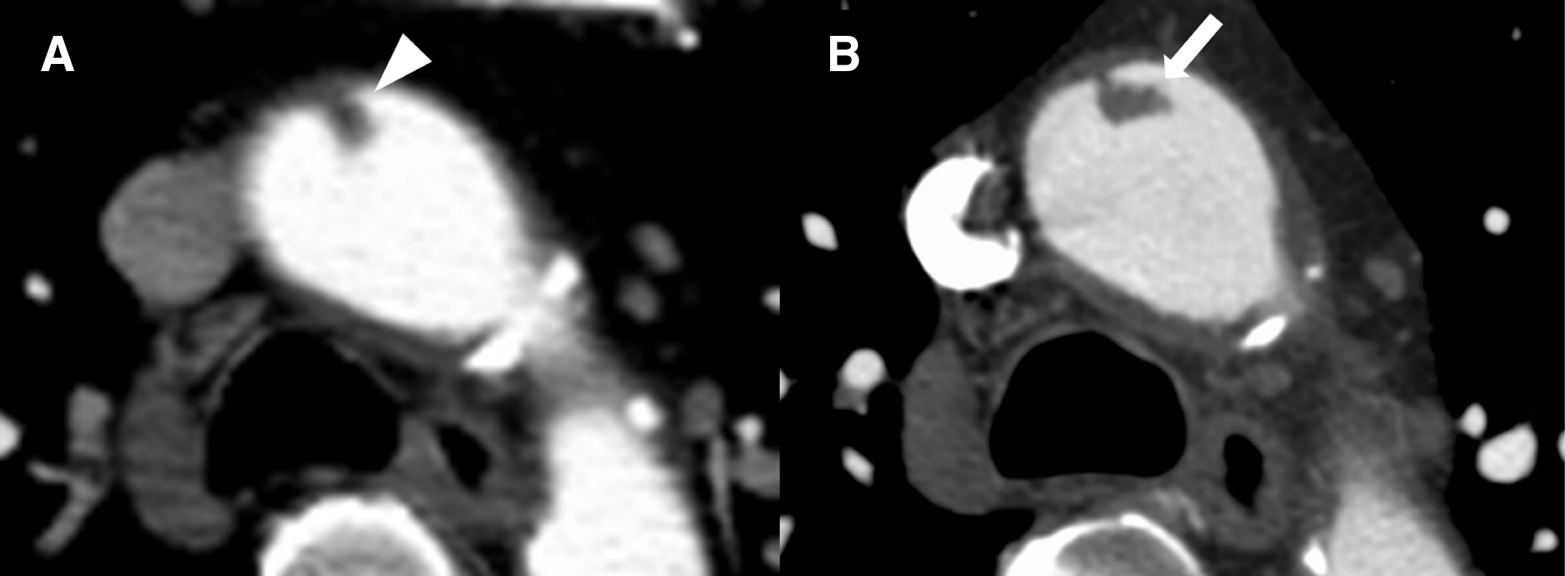
Preoperative contrast-enhanced CT findings. (**A**) Findings on the non-ECG-gated contrast enhanced CT revealed a small thrombus-like lesion that was identified in the ascending aorta, appearing relatively stable in nature (white arrowhead). (**B**) The ECG-gated contrast-enhanced CT provided clearer visualization of a highly mobile thrombus-like lesion with a thin stalk, reinforcing the indication for surgical treatment (white arrow). ECG, electrocardiogram

A 61-year-old man with a history of hypertension presented with sudden left hemiplegia. Emergency MRI revealed an embolic stroke in the right middle cerebral artery. The patient had no documented history of atrial fibrillation or other embolic sources. Although there was a history of acute lower limb arterial occlusion more than 10 years earlier, no underlying thrombophilic disorder, including antiphospholipid antibody syndrome, had been identified. His neurological symptoms improved during the examination, and he was admitted for conservative management. A non-ECG-gated contrast-enhanced CT performed after admission revealed a thrombus-like structure in the ascending aorta, prompting referral to our department (**Fig. 1A**). Although the initial CT suggested a relatively stable thrombus, an ECG-gated contrast-enhanced CT clearly demonstrated a thin-stalked and potentially mobile thrombus, leading to urgent surgical intervention (**Fig. 1B**).

The procedure was performed via median sternotomy. CPB was established using bilateral axillary arterial cannulation and bicaval venous drainage. To confirm that clamping of the LCCA would not reduce cerebral oxygen saturation, the artery was temporarily clamped before and after CPB initiation under NIRS monitoring. This strategy also helped prevent neurological complications during initiation of CPB. Preoperative MRA demonstrated an incomplete circle of Willis, with no communication between the anterior and posterior circulations, while robust bilateral communication was present within both the anterior and posterior circulations.

**Fig. 2:**
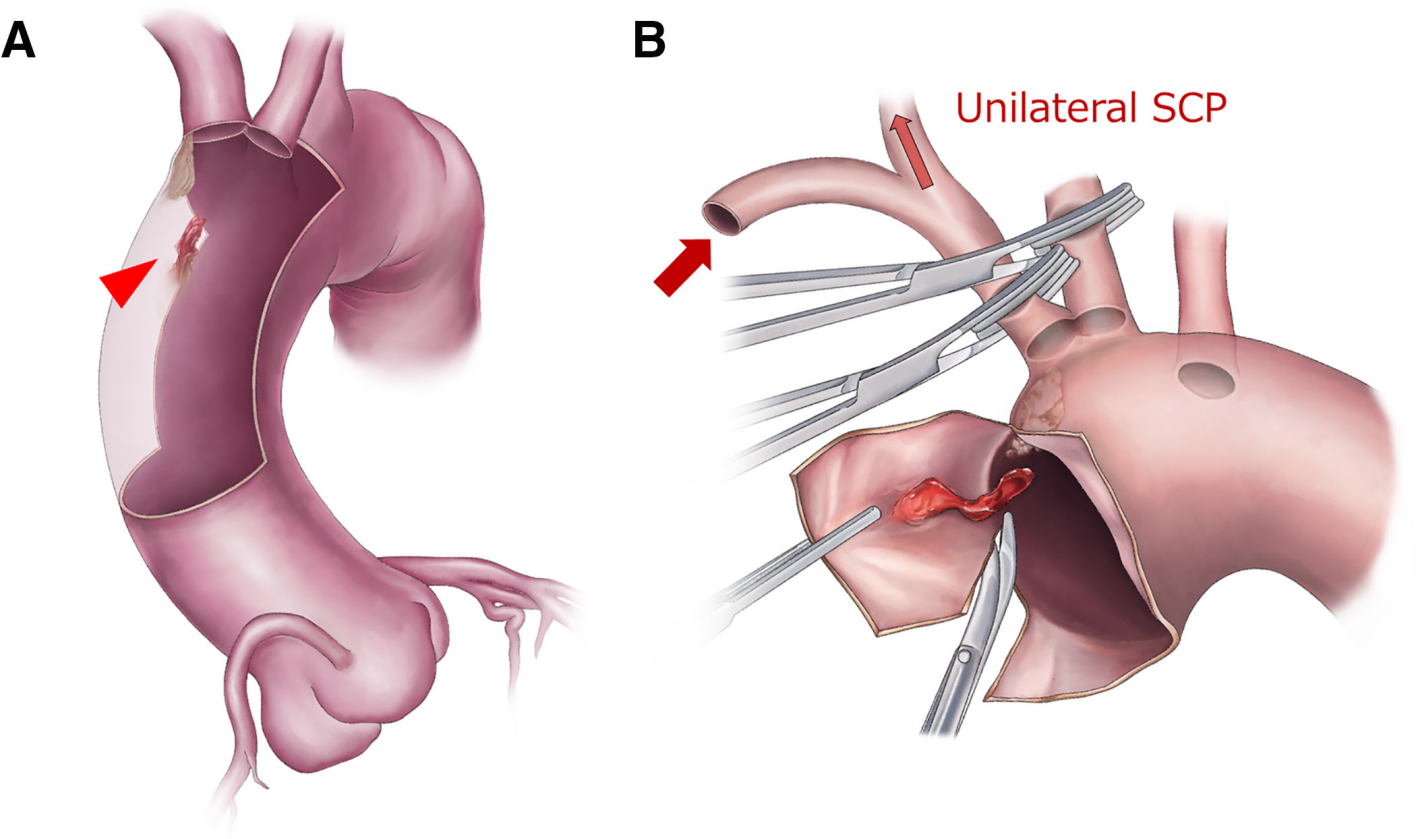
Schematic illustration of the surgical procedure. (**A**) The ascending aortic thrombus was located just proximal to the brachiocephalic artery (red arrowhead), with adjacent calcified lesions. The brachiocephalic artery and the left common carotid artery were in close proximity. (**B**) During establishment of cardiopulmonary bypass, the left common carotid artery was clamped, and during circulatory arrest the brachiocephalic artery was also clamped to achieve unilateral selective cerebral perfusion (unilateral SCP). The thrombus-like lesion was excised with an adequate margin. SCP, selective cerebral perfusion

After cooling to 26°C, circulatory arrest was initiated with clamping of the brachiocephalic artery and left common carotid artery. uSCP was employed at a flow rate of 600 mL/min (**Fig. 2**). Given the presence of a bovine aortic arch, a common trunk reconstruction of the brachiocephalic artery and left common carotid artery had initially been planned. Under this strategy, uSCP without left subclavian artery perfusion was considered sufficient. However, due to unsuitable arterial wall for reconstruction at the common trunk, the surgical strategy was modified intraoperatively to a two-branch reconstruction. As regional cerebral oxygen saturation monitored by NIRS remained stable, uSCP was continued throughout the procedure.

Upon opening the ascending aorta, a highly mobile thrombus-like mass was identified just proximally to the brachiocephalic artery. The lesion was excised with an adequate margin because a small possibility of malignancy could not be excluded. As the LCCA was in close proximity to the brachiocephalic artery, partial arch replacement with reconstruction of both arch branches was performed.

The aortic cross-clamp time was 63 min, and the circulatory arrest time was 39 min. The operation was completed without complications. Postoperative recovery was uneventful, with no new neurological deficits. After consulting with neurologists, the patient was discharged on POD 13, maintained on clopidogrel and warfarin for antithrombotic therapy. Pathology revealed atherosclerosis with fresh thrombi showing early organization, consistent with thrombotic etiology.

## DISCUSSION

We successfully performed surgery for this rare condition of ascending AMT. This case highlights two key clinical points. First, ECG-gated contrast-enhanced CT is essential for accurate diagnosis of ascending AMT. Second, a modified brain isolation strategy using uSCP allows safe surgical management without neurological complications.

While large ascending aortic thrombi described in previous reports generally pose little diagnostic uncertainty, smaller lesions—such as in this case—may easily be mistaken for motion artifacts. This suggests that similar cases may go unrecognized, underscoring the clinical relevance of this report. Although TEE was considered, the distal ascending aorta is a well-known “blind spot,” where visualization is hindered by interposed tracheal air.^5)^ In this patient, the non-ECG-gated CT suggested a small, stable thrombus, whereas the ECG-gated contrast-enhanced CT clearly demonstrated a thin stalk, strongly indicating the need for surgery. These findings support ECG-gated contrast-enhanced CT as essential when AMT is suspected.

Preventing neurological complications is crucial when operating for ascending AMT. The brain isolation technique was originally developed for high-risk cases—such as those with a shaggy aorta—undergoing arch surgery, where complete separation of cerebral circulation reduces embolic risk.^6,7)^ In this case, although achieving relatively stagnant flow in the ascending aorta via femoral artery perfusion may be considered, bilateral axillary artery cannulation combined with left common carotid artery occlusion was selected to prioritize cerebral protection at the initiation of CPB. This strategy was chosen to reduce the risk of cerebral embolization associated with thrombus dislodgement during CPB initiation, despite the thrombus being located near the brachiocephalic artery. Alternative perfusion strategies, including femoral artery perfusion alone or in combination with left axillary artery perfusion, may represent reasonable options in selected cases and warrant further consideration. Furthermore, using right axillary artery perfusion for uSCP minimized cerebral embolization and provided an uncluttered operative field, facilitating graft anastomosis. Although some studies suggest that uSCP provides outcomes comparable to bilateral SCP, including neurological results, uSCP may also reduce the risk of embolic events associated with additional cannulation.^8)^ However, there are limitations in determining the safety of uSCP based solely on NIRS monitoring. NIRS reflects only regional oxygenation of the superficial frontal cortex and does not adequately assess global cerebral perfusion, particularly in the posterior circulation, including the cerebellum and brainstem, as reported in previous studies.^9)^ Therefore, in select cases, bilateral selective cerebral perfusion may be required to ensure more definitive and comprehensive cerebral protection, and further investigation is warranted to clarify the indications for unilateral versus bilateral SCP.

## CONCLUSIONS

ECG-gated contrast-enhanced CT is the preferred diagnostic modality for ascending AMT. The CPB strategy used in this case—bilateral axillary artery cannulation with LCCA occlusion—together with the modified brain isolation technique using uSCP may provide an effective and safe approach for surgical treatment without neurological complications.

## DECLARATIONS

### Availability of data and materials

All data generated or analyzed during this case report are included in this published article. No additional datasets are available.
